# HECT, UBA and WWE domain containing 1 represses cholesterol efflux during CD4^+^ T cell activation in Sjögren’s syndrome

**DOI:** 10.3389/fphar.2023.1191692

**Published:** 2023-06-26

**Authors:** Junhao Yin, Jiabao Xu, Changyu Chen, Xinyi Ma, Hanyi Zhu, Lisong Xie, Baoli Wang, Yanxiong Shao, Yijie Zhao, Yu Wei, Anni Hu, Zhanglong Zheng, Chuangqi Yu, Jiayao Fu, Lingyan Zheng

**Affiliations:** ^1^ Department of Oral Surgery, Shanghai Ninth People’s Hospital, Shanghai Jiao Tong University School of Medicine, College of Stomatology, Shanghai Jiao Tong University, Shanghai, China; ^2^ National Center for Stomatology and National Clinical Research Center for Oral Disease, Shanghai, China; ^3^ Shanghai Key Laboratory of Stomatology, Shanghai, China; ^4^ Shanghai Institute of Stomatology, Shanghai, China; ^5^ Department of Oral and Maxillofacial Surgery, Shanghai Stomatological Hospital, Fudan University, Shanghai, China; ^6^ Department of Oral and Maxillofacial Surgery, Shanghai Engineering Research Center of Tooth Restoration and Regeneration, School and Hospital of Stomatology, Tongji University, Shanghai, China

**Keywords:** CD4^+^ T cell, HUWE1, ABCA1, Sjögren’s syndrome, cholesterol efflux

## Abstract

**Introduction:** Sjögren’s syndrome (SS) is a chronic autoimmune disorder characterized by exocrine gland dysfunction, leading to loss of salivary function. Histological analysis of salivary glands from SS patients reveals a high infiltration of immune cells, particularly activated CD4^+^ T cells. Thus, interventions targeting abnormal activation of CD4^+^ T cells may provide promising therapeutic strategies for SS. Here, we demonstrate that Hect, uba, and wwe domain containing 1 (HUWE1), a member of the eukaryotic Hect E3 ubiquitin ligase family, plays a critical role in CD4^+^ T-cell activation and SS pathophysiology.

**Methods:** In the context of HUWE1 inhibition, we investigated the impact of the HUWE1 inhibitor BI8626 and sh-Huwe1 on CD4^+^ T cells in mice, focusing on the assessment of activation levels, proliferation capacity, and cholesterol abundance. Furthermore, we examined the therapeutic potential of BI8626 in NOD/ShiLtj mice and evaluated its efficacy as a treatment strategy.

**Results:** Inhibition of HUWE1 reduces ABCA1 ubiquitination and promotes cholesterol efflux, decreasing intracellular cholesterol and reducing the expression of phosphorylated ZAP-70, CD25, and other activation markers, culminating in the suppressed proliferation of CD4^+^ T cells. Moreover, pharmacological inhibition of HUWE1 significantly reduces CD4^+^ T-cell infiltration in the submandibular glands and improves salivary flow rate in NOD/ShiLtj mice.

**Conclusion:** These findings suggest that HUWE1 may regulate CD4^+^ T-cell activation and SS development by modulating ABCA1-mediated cholesterol efflux and presents a promising target for SS treatment.

## 1 Introduction

Sjögren’s syndrome (SS) is a chronic autoimmune disorder characterized by abnormal exocrine gland function (Mavragani and Moutsopoulos, 2014). System damage, such as renal tubular acidosis, hypothyroidism, and liver damage, can often accompany SS. Local lesions found in patients with SS are similar to those of other autoimmune diseases such as rheumatoid arthritis and systemic scleroderma, which are characterized by CD4^+^-T cell hyperactivation and proliferation (Sharabi and Tsokos, 2020), (Ryu et al., 2019). Uncontrolled CD4^+^-T cell hyperactivation can trigger aberrant immune responses of B cells and the recruitment of neutrophils, further exacerbating the disease. Thus, targeting CD4^+^-T cell hyperactivation may provide a promising therapeutic approach for SS.

Auto-activated T cells have been shown to undergo abundant metabolic reprogramming, from the elevated transcription of metabolic genes to the production of metabolites during the development of autoimmunity (Buck et al., 2015). Recent studies have demonstrated the critical role of cholesterol metabolism in T-cell biology (Liu et al., 2021). As an important component of cellular membranes, cholesterol helps to maintain plasma membrane stability and modulate its fluidity (King et al., 2022). Additionally, cholesterol is involved in the formation of key structures, such as lipid rafts, major histocompatibility complex molecules, and T-cell receptors, which are integral to adaptive immunity (Montixi et al., 1998), (Hiltbold et al., 2003). Recent studies have demonstrated that the upregulation of membrane cholesterol promotes TCR nanoclustering and the formation of immunological synapses, thereby enhancing T-cell proliferation (Yang et al., 2016), (Molnár et al., 2012). Cholesterol molecules can also bind to the TCR complex in its resting state, conferring conformational restriction; this can be released upon antigen binding and subsequent TCR activation (Chen et al., 2022).

Cholesterol homeostasis in immune cells is governed by four processes: biosynthesis, efflux, absorption, and storage. Among these, cholesterol efflux is mediated by several ATP-binding cassette transporters, including ATP-binding cassette sub-family A member 1 (ABCA1) and ATP-binding cassette sub-family G member 1 (ABCG1), allowing cells to remove excess cholesterol (Luo et al., 2020; Qin et al., 2020). Notably, activated CD4^+^ T cells immediately downregulate the expression of cholesterol efflux transporters to preserve newly synthesized cholesterol (Michaels et al., 2021), while upregulated ABCG1 suppresses T-cell proliferation (Bensinger et al., 2008). However, the explicit molecular mechanisms that regulate the expression of these molecules and the subsequent cholesterol homeostasis that occurs upon T-cell activation are still largely unelucidated.

Over the last decade, research has suggested that the ubiquitous regulatory network may be involved in cholesterol homeostasis and immune responses (Hu et al., 2021), (van Loon et al., 2020). The ubiquitin-proteasome system is a major physiological mechanism for protein degradation in cells, responsible for the degradation of over 80% of proteins. This intricate system involves the dynamic post-translational modification of proteins by three enzymes: ubiquitin-activating enzyme (E1), ubiquitin-conjugating enzyme (E2) and ubiquitin-protein ligase (E3). Ubiquitin ligases are essential proteins for recognizing substrates and determining the specificity of ubiquitination reactions. For example, sterol-induced ubiquitination and proteasome degradation of HMGCR, an important feedback mechanism of cellular regulation of cholesterol homeostasis, are synergized by ubiquitin-ligase gp78 (Song et al., 2005), Trc8 (Jo et al., 2011) and MARCH6 (Zelcer et al., 2014), (Scott et al., 2020). Ubiquitination of the low-density lipoprotein receptor (LDLR) is modulated by ubiquitin ligase Idol (Zelcer et al., 2009), (Loregger et al., 2016). Ubiquitin ligase HECTD1 regulates the stability of ATP-binding cassette transporter A1 (ABCA1) to affect cholesterol export (Aleidi et al., 2018). However, the role of the ubiquitin-proteasome system in the regulation of cholesterol metabolism in T cells remains to be elucidated.

In this study, we report on our findings that *HUWE1* is highly expressed in peripheral blood CD4^+^ T cells of SS patients. Similarly, we found that *Huwe1* expression is significantly upregulated in activated murine CD4^+^ T cells. *In vitro* experiments showed that inhibition of HUWE1 function led to a decrease in CD4^+^ T cell activation and proliferation and a decrease in cytoplasmic membrane cholesterol content. Furthermore, inhibition of HUWE1 promoted cholesterol efflux without affecting cholesterol synthesis, and decreased the ubiquitination level of ABCA1, resulting in increased stability. Pharmacological inhibition of HUWE1 also significantly improved SS-like autoimmunity in NOD/ShiLtj mice. Our study contributes to the understanding of the regulatory mechanisms underlying ubiquitination and cholesterol homeostasis in CD4^+^ T cells, offering new insights for the development of more effective treatments for SS.

## 2 Materials and methods

### 2.1 Raw data collection and functional enrichment analysis

Two recently published datasets from the NCBI GEO (Edgar et al., 2002) contain gene expression profiles obtained from the labial glands and CD4^+^ T cells of SS patients and healthy donors. Specifically, we analyzed 18 patients with SS and 18 healthy controls in the salivary gland dataset (accession number: GSE94510) (Tasaki et al., 2017) and 17 SS patients and 15 healthy controls in the CD4^+^-T cell dataset (accession number: GSE143153) (Joachims et al., 2020). These datasets provide an invaluable resource to further explore the molecular mechanisms that underpin SS. The expression data were processed using the IOBR package in R for conversion into TPM format (Zeng et al., 2021). Additionally, Gene ontology-biological process (GO-BP) gene sets were obtained using the msigdbr package (Liberzon et al., 2015), (Subramanian et al., 2005) as described (Yin et al., 2020), (Yin et al., 2022a), (Yin et al., 2022b). The corresponding web links are provided in Supplementary File S1.

### 2.2 Acquisition of the study samples

This study was approved by the Ethics Committee of the Shanghai Ninth People’s Hospital affiliated to the Shanghai Jiao Tong University School of Medicine (Approval IDs: SH9H-2019-T159-2 and SH9H-2021-TK69-1). All human participants provided written informed consent. The selected SS patients met the criteria of the American-European Consensus Group for SS (Bruserud, 2001) and had not received any immunosuppressive or immunomodulatory drugs before sample collection. All donors were middle-aged females aged 30–60 years old. Peripheral blood was collected from each donor (10 mL) and peripheral blood mononuclear cells (PBMCs) were isolated as described (Fu et al., 2020). Then, human CD4^+^ T cells were isolated from the PBMCs by the Human CD4^+^ T Cell Enrichment Kit (19,052, Stemcell Technologies) according to the manufacturer’s instructions. After suspension of PBMCs obtained with a 37 μm cell strainer (Falcon), a cell suspension of approximately 5 × 10^7 cells/mL was prepared in PBS containing 2% (v/v) fetal bovine serum and 1 mM EDTA. Subsequently, 50 μL/mL of Enrichment Cocktail was added and the cells were cultured for 10 min. A magnetic bead solution (100 μL/mL) was then added to the mixture for 5 min, after which the round-bottom test tube was placed in a magnet. The cell suspension was emptied from the tube after 5 min and used for subsequent experiments.

### 2.3 Animals

Female C57BL/6 mice and NOD/ShiLtj mice were purchased from the Model Animal Research Center of Nanjing University (China), and were cared for in accordance with the criteria outlined in the Guide for the Care and Use of Medical Laboratory Animals (National Research Council, 2011). BI8626 (HY-120204, MCE, 44 mg/kg) and vehicle [40% (v/v) PEG300, 5% (v/v) Tween-80, 45% (v/v) saline] were administered to the experimental group and control group, respectively. At 8 weeks of age, mice were intraperitoneally injected with 3.6 mg/mL BI8626 twice or four times a week for 4 weeks. In the control group, mice were administered an intraperitoneal injection of vehicle (200–250 μL) twice weekly for 4 weeks. Once weekly, salivary flow rate (SFR) was measured in mice by an intraperitoneal injection of pilocarpine (5 mg/kg, HY-B0726, MCE) to induce saliva production. A pipette tip was placed in the mouth of the animals, and saliva was collected for 15 min to calculate the SFR. Following cervical dislocation, the bilateral salivary gland tissues of the mice (12 weeks) were obtained. Hematoxylin & eosin (H&E) staining was performed on one side of each mouse’s salivary gland tissue, and the severity of lymphocyte infiltration in the gland tissues was evaluated using a classical scoring system (Chisholm et al., 1970), (Gu et al., 2018). An aggregate of more than fifty lymphocytes in a random field (4 mm^2^) of the salivary gland tissue was defined as a lymphocytic focus, according to the score system detailed in Table 1. Five mice per group were used as biological replicates in a study designed to assess the presence of lymphocytic foci in salivary gland tissue. Tissue samples were minced and then digested with 1 mg/mL collagenase type 4 and DNase I for 45 min. The resulting suspension was then passed through a 70 μm mesh nylon strainer (Falcon) and rinsed with PBS containing 2% (v/v) fetal bovine serum (FBS, Gibco).

**TABLE 1:** The standards of the scoring system.

Grade	Description	Lymphocytic foci per 4 mm^2^
0	None	0–1 lymphocyte
1	Slight infiltrate	2–8 lymphocytes
2	Moderate infiltrate	9–50 lymphocytes
3	One focus	>50 lymphocytes
4	More than one focus	>100 lymphocytes

Following centrifugation, cells were resuspended in 3 mL PBS supplemented with 37% (v/v) Percoll, and carefully layered on top of 3 mL of PBS containing 70% (v/v) Percoll. An additional 3 mL of PBS containing 30% (v/v) Percoll was overlaid, and the sample was centrifuged at 800 *g* for 20 min. The cell layer was collected, resuspended in PBS, and further purified. Cells were labeled with PE-Cy7 anti-mouse CD45 (#561868, BD Biosciences), FITC anti-mouse CD3 (#100204, BD Biosciences), PE anti-mouse CD4 (#100512, BD Biosciences), and APC anti-mouse CD8 (#100711, BD Biosciences) for 30 min. Subsequently, the fluorescence intensity was measured using a Beckman CytoFlex S.

### 2.4 Cell culture

Spleens were carefully ground in a 70 μm mesh nylon strainer (Falcon) and rinsed with PBS containing 2% (v/v) FBS to obtain a single-cell suspension. We incubated the centrifuged cell masses with 5 mL erythrocyte lysate on ice for 2 min, followed by a second centrifugation. Subsequently, total CD4^+^ T cells were isolated from the cell suspension using a Mouse CD4^+^ T Cell Positive Selection Kit (18,952, Stemcell Technologies). We suspended cell masses in 1 × 10^8 cells/mL PBS containing 2% (v/v) FBS and 1 mM EDTA and then added 50 μL/mL rat serum and 50 μL/mL Selection Cocktail for 5 min. Subsequently, 30 μL/mL magnetic bead solution was added for 3 min, followed by 1.5 mL PBS containing 2% FBS and 1 mM EDTA. The round-bottom test tube containing the mixture was then placed in a magnet for 3 min, then the cells were washed twice with the previously described PBS solution. The purified cells and magnetic beads were left in the test tube for further use. The purified cells were then cultured in Rosewell Park Memorial Institute (RPMI) 1640 medium (Gibco) supplemented with 10% (v/v) FBS and 1% (v/v) penicillin/streptomycin (HyClone), and stimulated with a combination of 5 μg/mL plate-bound anti-CD3ε and anti-CD28 antibodies (BD Biosciences). Chemical inhibitors included BI8626 (HY-120204, MCE), N-Butyldeoxynojirimycin (NB-DNJ, UGCG inhibitor, HY-17020, MCE), and DIDS (HY-D0086, MCE).

### 2.5 Proliferation assay

Using the highly stable cell tracer 5-(and-6)-Carboxyfluorescein Diacetate Succinimidyl Ester (CFSE)-based cell proliferation kit (C34554, Thermo Fisher Scientific), we assessed the degree of T cell proliferation. We incubated CD4^+^ T cells in suspension with 2 μM CFSE solution in the dark for 10 min. To stop the staining, 10 mL of medium containing 10% (v/v) FBS was added to the cell suspension and incubated on ice for a further 10 min in the dark. After staining, the cells were used in subsequent experiments. We then monitored the fluorescence intensity using a Beckman CytoFlex to detect changes in cell proliferation.

### 2.6 Cell cycle analysis

Analysis of the cell cycle phase distribution was conducted using propidium iodide (PI) staining of DNA content. Cells were first washed with pre-cooled PBS and centrifuged, then fixed with 70% ethanol at −20°C for 2 h. After removing the ethanol, cells were washed with PBS and mixed with a working solution containing 25 μL PI solution and 2.5 μL RNase. Staining was done at 37°C for 30 min, followed by incubation at 4°C for an additional 30 min. Cell clumps were removed from the sample by filtration through a 70 μm nylon screen and DNA quantification was conducted using a Beckman CytoFlex.

### 2.7 Filipin III staining and cellular cholesterol determination

T cells were seeded into a confocal-private plate using Cell-Tak cell and tissue adhesive (Corning), and then fixed with 4% (w/v) paraformaldehyde. Intracellular cholesterol was fluorescently stained using a cholesterol cell-based detection assay kit (Cayman) for 30 min at 4°C in the dark. Images of stained cells were acquired on an ECLIPSE Ts2R laser-scanning confocal microscope (Nikon). Total cholesterol was extracted and determined using a total cholesterol detection kit (Applygen Technologies), while cellular protein content was measured with an Enhanced BCA Protein Assay Kit (Beyotime).

### 2.8 Cholera toxin B staining assay

Cells were fixed with 4% (w/v) polyformaldehyde for 30 min at room temperature, and then incubated with a biotin-labeled cholera toxin B subunit conjugated with FITC (C1655, Sigma-Aldrich). Subsequent analysis by the Beckman CytoFlex S enabled the characterization of lipid rafts.

### 2.9 TCR signaling

Splenocytes were first stimulated with 1 μg/mL α-CD3 antibody for up to 90 min at 37°C. Cells were then fixed with 4% paraformaldehyde and permeabilizated in ice-cold 100% methanol. Subsequently, antibodies against phosphorylated Zap70 (683705, Biolegend) were used for immunostaining. This approach allowed us to elucidate the dynamics of TCR signaling.

### 2.10 Cholesterol efflux assay

Cholesterol efflux detection was based on Zhao et al. (Zhao et al., 2022). Purified murine CD4^+^ T cells from the relevant groups were labeled with a medium containing 2.5 mM BODIPY-cholesterol (HY-125746, MCE), obtained by complexing the sterols (unlabeled cholesterol and BODIPY-cholesterol, 8:2) with methyl-β-cyclodextrin (HY-101461, MCE) at a molar ratio of 1:15. Labeling was carried out by incubating the cells in labeling medium supplemented with 0.2% fatty acid-free BSA and 2 μg/mL Sandoz58-035 (ACAT inhibitor), at 37°C and 5% CO_2_ for 60 min. After washing twice with normal culture medium and centrifuging at 350 g for 5 min, the cells were resuspended in culture medium for a 1 h equilibration. ApoAI (10 μg/mL, A0722, Sigma-Aldrich) or HDL (50 μg/mL, SAE0054, Sigma-Aldrich) was then added to the cell suspension to initiate cholesterol efflux. After a four-hour incubation period, efflux was stopped by centrifuging at 350 g for 5 min to remove the supernatant-containing acceptors. Samples treated without ApoAI or HDL after equilibration were used as the baseline for efflux. The mean fluorescence intensity (MFI) of BODIPY-cholesterol on T cells was subsequently measured by flow cytometry. The level of cholesterol efflux was then calculated as follows: (baseline MFI-remaining MFI)/baseline MFI×100.

### 2.11 Western blotting (WB)

Protein extraction was performed using a Pierce immunoprecipitation (IP) lysis buffer (Thermo Fisher) supplemented with a Protease Inhibitor Cocktail (Thermo Fisher). Samples were separated by SDS-PAGE gel electrophoresis and transferred to a PVDF membrane (Millipore). The membrane was blocked with Quick Block Buffer (Beyotime Biotechnology) for 15 min and then treated overnight with primary antibodies against Cyclin-dependent kinase 2 (CDK2, 1:1000, CST), Cyclin-dependent kinase 4 (CDK4, 1:1000, CST), Hydroxymethylglutaryl-CoA reductase (HMGCR, 1:1000, CST), Squalene epoxidase (SQLE, 1:1000, CST), HMGCS1 (1:1000, CST), ATP-binding cassette transporter A1 (ABCA1, 1:1000, Abcam), ATP-binding cassette transporter G1 (ABCG1, 1:1000, Abcam), Scavenging Receptor SR-BI (SR-BI, 1:1000, Abcam), HUWE1 (1:1000, Abcam) and ACTIN (1:1000, CST) at 4°C. After incubation with the primary antibody, the membrane was allowed to return to room temperature over 1 h. Subsequently, the membrane was washed three times with 1×TBST buffer for 10 min each time. Horseradish peroxidase (HRP)-labeled goat anti-rabbit IgG (H + L) (Beyotime) or horseradish peroxidase (HRP)-labeled goat anti-mouse IgG (H + L) (Beyotime) was then diluted with 1×TBST buffer and incubated with the membrane at room temperature for 2 h, followed by three washes with 1×TBST. Finally, Immobilon Western Chemiluminescent HRP Substrate (Millipore) was used. The membrane was then blotted on filter paper and incubatd in full contact with luminescent working liquid at room temperature for 1 min. The results of the WB analysis were visualized and quantified using an Amersham Imager 600.

### 2.12 Real-time quantitative polymerase chain reaction (RT-qPCR)

Total RNA was extracted from T cells with TRIzol Reagent (TaKaRa) according to the manufacturer’s protocol (Yin et al., 2022a). Chloroform (0.2 mL) was added to 1 mL of cell lysate and mixed by shaking. The mixture was then placed on ice for 15 min, centrifuged, and the uppermost clear phase was collected. An equal volume of isopropyl alcohol was then added to the mixture and the mixture was allowed to stand for 10 min before a second centrifugation. The liquid in the tube was discarded, and 0.6 mL of pre-cooled 75% (v/v) ethanol was added to wash the RNA precipitates on the tube wall. This process was repeated twice, after which the ethanol was discarded. When the edges of the RNA precipitate became clear, it was dissolved in 20 μL DEPC water and the concentration and purity of the dissolved precipitate were measured using a Biochrom NanoVue Plus. All steps were carried out on ice. Subsequently, 1000 ng of total RNA was reverse-transcripted to cDNA using Takara PrimeScript RT reagent kits (TaKaRa), which was then used for RT-qPCR on a LightCycler96 Instrument (Roche). The primer sequences are listed in Table 2, and *ACTB/Actb* was used as the internal mRNA control. Experiments were repeated in triplicate, and the relative RNA expression rates were calculated using the 2^-△△Ct^ method.

**TABLE 2:** A list of Primers.

Gene and primer type	Primer sequences (5′to 3′)
*Huwe1*	
Forward primer	GAG​GGC​GTA​AAC​ATA​CAG​AGA​AG
Reverse primer	CGC​TGC​TGT​GTA​AAG​TGG​C
*Hmgcr*	
Forward primer	TCT​TGT​GGA​ATG​CCT​TGT​GAT​T
Reverse primer	GGG​TTA​CGG​GGT​TTG​GTT​TAT
*Hmgcs1*	
Forward primer	CGA​ACC​CTC​CTC​AAG​AAG​CC
Reverse primer	CCT​AGA​ACA​CAG​AGA​TTC​CGG​C
*Sqle*	
Forward primer	GCT​GGG​CCT​TGG​AGA​TAC​AG
Reverse primer	CAG​TGG​GTA​CGG​AAT​TTG​AAC​T
*Cd69*	
Forward primer	AAG​CGA​TAT​TCT​GGT​GAA​CTG​G
Reverse primer	ATT​TGC​CCA​TTT​CCA​TGT​CTG​A
*Cd25*	
Forward primer	AAC​CAT​AGT​ACC​CAG​TTG​TCG​G
Reverse primer	TCC​TAA​GCA​ACG​CAT​ATA​GAC​CA
*Il-2*	
Forward primer	GTG​CTC​CTT​GTC​AAC​AGC​G
Reverse primer	GGG​GAG​TTT​CAG​GTT​CCT​GTA
*Nr1h3*	
Forward primer	CTC​AAT​GCC​TGA​TGT​TTC​TCC​T
Reverse primer	TCC​AAC​CCT​ATC​CCT​AAA​GCA​A
*Abca1*	
Forward primer	AAA​ACC​GCA​GAC​ATC​CTT​CAG
Reverse primer	CAT​ACC​GAA​ACT​CGT​TCA​CCC
*Abcg1*	
Forward primer	CTT​TCC​TAC​TCT​GTA​CCC​GAG​G
Reverse primer	CGG​GGC​ATT​CCA​TTG​ATA​AGG
*Scarb1*	
Forward primer	TTT​GGA​GTG​GTA​GTA​AAA​AGG​GC
Reverse primer	TGA​CAT​CAG​GGA​CTC​AGA​GTA​G
*Actb*	
Forward primer	GAT​CAA​GAT​CAT​TGC​TCC​TCC​TG
Reverse primer	AGG​GTG​TAA​AAC​GCA​GCT​CA
*HUWE1*	
Forward primer	CCA​GAA​GTT​CTT​CTT​GAG​GGT​ACT
Reverse primer	GCCTAAACCGGAGGAACC
*ACTB*	
Forward primer	TGG​CAC​CCA​GCA​CAA​TGA​A
Reverse primer	CTA​AGT​CAT​AGT​CCG​CCT​AGA​AGC​A

### 2.13 Short hairpin RNA (shRNA) transfection

Huwe1-shRNA lentivirus (shRNA-*Huwe1*-01, shRNA- *Huwe1*-02 and shRNA- *Huwe1*-03) and normal control lentivirus were designed and synthesized by Hanbio (Shanghai). These shRNAs were transfected into murine CD4^+^ T cells according to the manufacturer’s recommended protocol (Li et al., 2018).

### 2.14 Statistical analysis

Statistical analyses were performed using GraphPad Prism 8.0 software. Data are presented as means±standard deviations (SDs). Student’s t tests were used to compare groups, with *p* < 0.05 indicating a statistically significant difference. A one-way analysis of variance followed by Tukey’s multiple comparison *post-hoc* tests was employed for multiple comparisons.

## 3 Results

### 3.1 *Huwe1* is positively related to CD4^+^ T cell activation and the occurrence of SS

**FIGURE 1:**
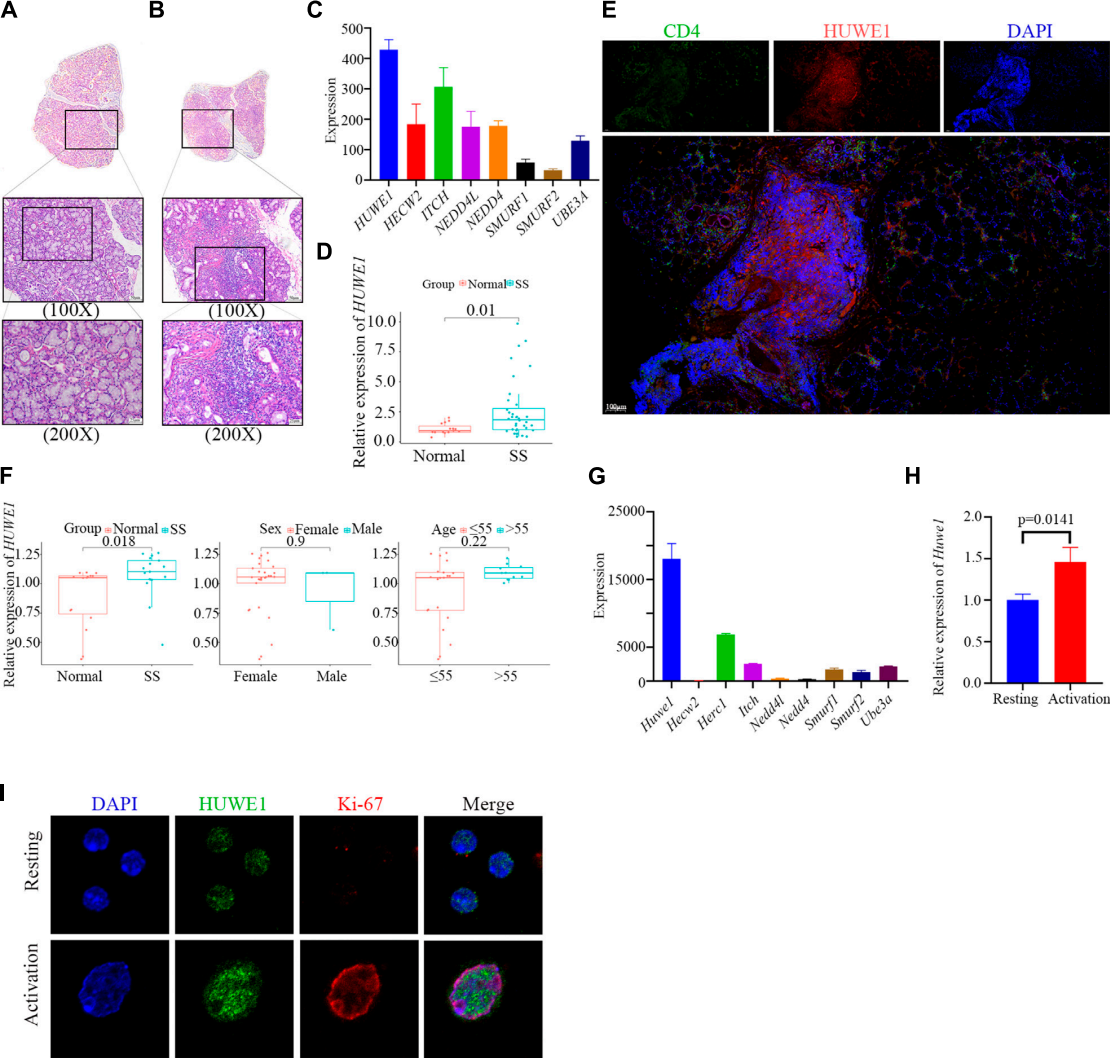
HUWE1 is positively related to CD4^+^ T cell activation and the occurrence of Sjögren syndrome. **(A,B)** Lymphocyte infiltration was detected by H&E staining of the labial salivary glands of normal individuals **(A)** and patients with pSS **(B)**. **(C)** Ubiquitin ligase gene expression in labial glands from SS patients based on sequencing data. The expression of HUWE1 is higher than other E3 ligase genes. **(D)** Expression of HUWE1 in circulating CD4^+^ T cells obtained from 5 healthy individuals and 11 SS patients. The expression of each gene was normalized to β-ACTIN expression. Three independent experiments were performed for qRT-PCR assays. **(E)** Immunofluorescence staining of CD4-positive cells and HUWE1-positive cells in the labial glands of SS patients. There are more CD4^+^ T cells in the positive HUWE1 region than in the negative HUWE1 region. **(F)** Correlation analysis between HUWE1 expression and clinical characteristics. The gene profile of GSE143153 is based on CD4^+^ T cells derived from labial salivary glands of SS patients and healthy donors. **(G)** Ubiquitin ligase gene expression in murine CD4^+^ T cells upon activation based on sequencing data. The expression of Huwe1 is higher than other E3 ligase genes. **(H)** Murine CD4^+^ T cells were cultured in the presence of 5 μg/mL plate-bound anti-CD3ε and 2 μg/mL anti-CD28 for 48 h. The expression of Huwe1 was evaluated by real-time PCR. The expression was normalized to β-actin expression and normalized relative to expression in CD4^+^ T cells at 0 h. Data shown are means ± SD. Data are from one experiment representative of three independent experiments with similar results. **(I)** Immunofluorescence was performed to detect the expression of HUWE1 in resting and activated CD4^+^ T cells. DAPI was used to stain the nuclei and glowed blue. Ki-67 was red light, and HUWE1 was green light.

Salivary glands from SS patients were characterized by extensive lymphocyte infiltration, which was also present in the samples we collected (Figures 1A–C). We further isolated CD4^+^ T cells from the peripheral blood of SS patients and conducted PCR verification. We found higher expression of *HUWE1* in CD4^+^ T cells of the SS group (Figure 1D). Furthermore, immunohistochemical and immunofluorescence staining were performed on labial gland samples from SS patients. Immunohistochemical results showed a possible overlap between the CD4 positive area and the HUWE1 positive area (Supplementary File S2A). Immunofluorescence further confirmed an increased presence of CD4^+^ T cells in the positive HUWE1 region, as compared to the negative HUWE1 region (Figure 1E; Supplementary File S2B). In our analysis of the gene microarray dataset (GSE143153) from the GEO database, we identified an increased expression of *HUWE1* in CD4^+^ T cells derived from labial glands of SS patients, compared to healthy controls (Figure 1F). These latter findings provide an external validation of the observed upregulation of *HUWE1* in SS. However, no significant correlation was observed between *HUWE1* expression and clinical characteristics, such as age and sex (Figure 1F). Using splenic CD4^+^ T cells from mice, we examined the expression of ubiquitin ligase genes in activated cells by gene sequencing (Yin et al., 2022c). As shown in Figure 1G, *Huwe1* was highly expressed in activated murine CD4^+^ T cells. The results of PCR assays showed that *Huwe1* expression was significantly upregulated in CD4^+^ T cells after activation (Figure 1H). Immunofluorescence staining also showed that HUWE1 expression was significantly increased in activated versus resting CD4^+^ T-cells (Figure 1I). This further supports the hypothesis that HUWE1 may play an important role in CD4^+^T cell infiltration foci.

### 3.2 *Huwe1*-deficient CD4^+^ T cells display impaired TCR signaling, reduced proliferation, and increased apoptosis upon activation

**FIGURE 2:**
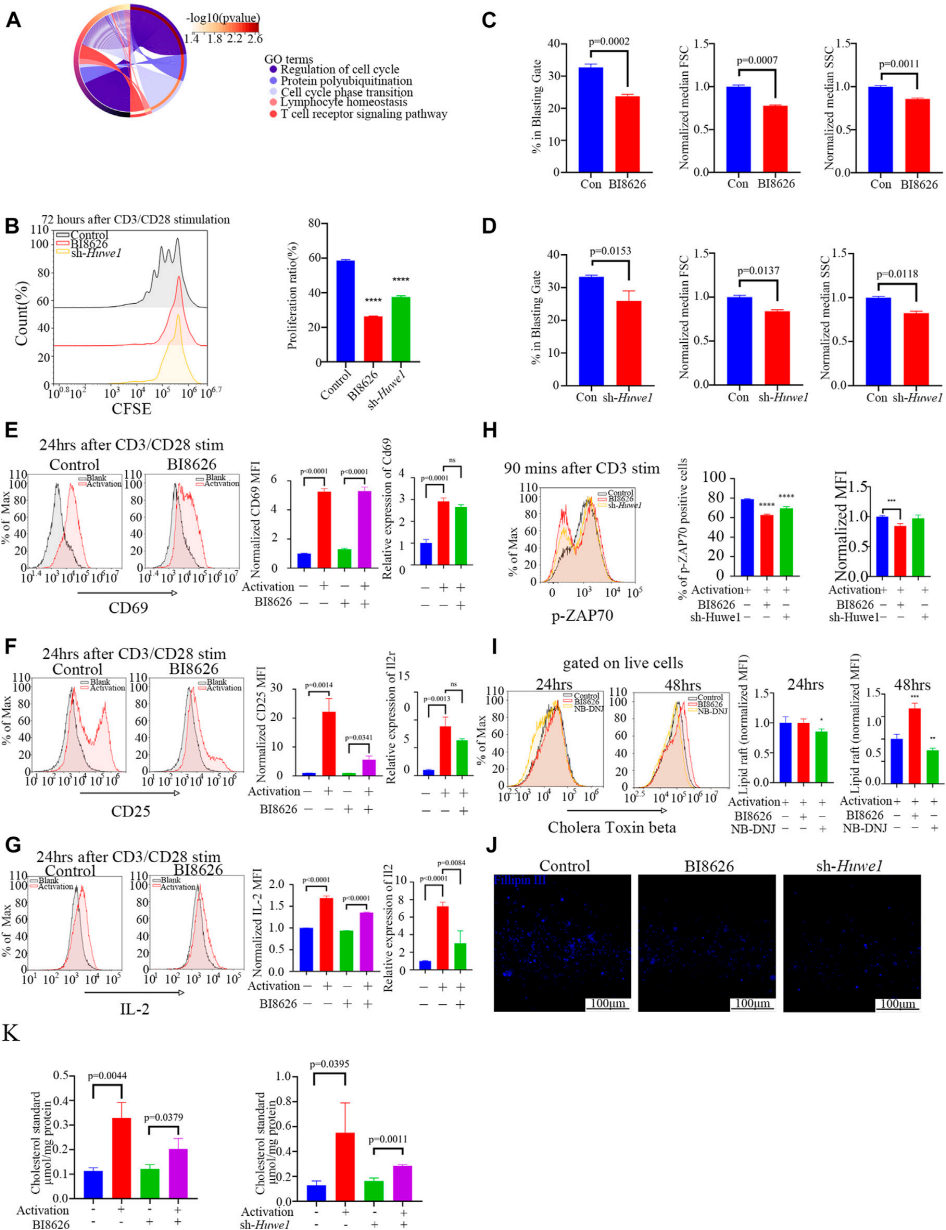
*Huwe1*-deficient CD4^+^ T cells display impaired TCR signaling, reduced proliferation, and increased apoptosis after activation **(A)** Biological processes enriched by Gene Ontology analysis of differentially expressed genes between SS patients with high *Huwe1* expression and low *Huwe1* expression. **(B)** Representative flow cytometry images of 5,6-carboxyfluorescein diacetate, succinimidyl ester (CFSE)-based fluorescence intensity in the indicated groups. The proliferation proportion of CD4^+^ T cells was downregulated under treatment of BI8626 and sh-Huwe1. Quantitation of proliferation proportion of CD4^+^ T cells as indicated. Quadruple asterisks indicate *p* < 0.0001 vs. control. **(C)** to **(D)** Cell blasting of BI8626-treated **(C)** and sh-Huwe1-treated **(D)** CD4+T cells was analyzed by size (forward scatter area, FSC) and granularity (side scatter area, SSC) after 24h activation. Bar graphs represent mean ± SD. **(E–G)** Enriched CD4+T cells were stimulated with anti-CD3ε and anti-CD28 in the presence or absence of BI8626 and analyzed for CD69 **(E)**, CD25 **(F)** and interleukin-2 (IL-2) **(G)** expression after 24 h. Bar graph on right shows MFI±SD. **(H)** Enriched CD4^+^ T cells were activated in the presence of BI8626 or sh-Huwe1 and analyzed for phosphorylated-ZAP70 expression after 90 min. Bar graph on right shows MFI±SD. Triple asterisks indicate *p* < 0.001, quadruple asterisks indicate *p* < 0.0001 vs. control. **(I)** Representative histograms showing levels of glycosphingolipid expression, determined by CTB staining, on membranes of purified splenic CD4^+^ T cells in the indicated groups. Asterisk indicates *p* < 0.05, double asterisks indicate *p* < 0.01, triple asterisks indicate *p* < 0.001 vs. control. **(J)** Total CD4+T cells were cultured in the presence of 5 μg/mL plate-bound anti-CD3ε and 2 μg/mL anti-CD28 for 48 h. Three indicated groups of T cells were purified, stained with Fillipin Ⅲ, and then analyzed for cholesterol content by fluorescence microscopy. Scale bars, 100 μm. **(K)** Total cholesterol from different treatment groups were subjected to a total cholesterol detection kit. The experiment was repeated twice, and representative images are shown. Data are from one experiment representative of three **(B–G)**; (*n* = 3) or two **(H,I)**; (*n* = 3) independent experiments with biological duplicates in each.

We obtained sequence data of labial salivary gland tissues (GSE94510) from SS patients from the NCBI GEO database to define the functions of *HUWE1*. These data provide insight into the role of *HUWE1* in SS pathogenesis (Edgar et al., 2002). We interrogated eighteen SS samples divided into two groups based on the *HUWE1* expression level, and performed a functional enrichment analysis to infer biological functions of DEGs between the two groups. Functional analysis revealed a role for *HUWE1* in T-cell receptor signaling pathway, lymphocyte homeostasis, and protein polyubiquitination (*p*-value < 0.05, Figure 2A). These findings indicated that *HUWE1* is likely to be involved in the regulation of T-cell activation. We then tested the effect of HUWE1 on CD4^+^ T cell activation by treating murine splenic CD4^+^ T cells with the selective HUWE1 inhibitor BI8626 (Peter et al., 2014) and sh-*Huwe1* (Supplementary Files S2C, D). Murine CD4^+^ T-cell proliferation was inhibited by BI8626 and sh-*Huwe1*, as shown in CFSE proliferation assays (Figure 2B). Following anti-αCD3/CD28 stimulation, CD4^+^ T cells treated with BI8626 or sh-*Huwe1* exhibited a decreased frequency of blasts and a significantly reduced median size, as measured by the forward scatter area (FSC) on the flow cytometric analyses, compared to the control group (Figures 2C, D). Huwe1-deficient CD4^+^ T cells also exhibited reduced granularity as evidenced by a marked decrease in side scatter area (SSC). In addition, no significant change in overall apoptosis level of cells was observed across different treatment groups (Supplementary File S2E). To further investigate cell cycle changes influenced by cell proliferation, flow cytometry was used to analyze cell cycle distributions in cells treated with BI8626 or sh-*Huwe1*. Results showed an increased number of cells in the S phase in the BI8626 or sh-*Huwe1* treatment groups (Supplementary File S2F). This was accompanied by a significant reduction in the expression of CDK2, a key regulator of S phase progression (Supplementary File S2G). To investigate the underlying mechanism, we examined T-cell activation. Analysis of three activation biomarkers-CD69, CD25, and IL-2 in CD4^+^ T cells following treatment with BI8626 revealed a non-significant decrease in the CD69 positive rate and Cd69 mRNA expression. Mean fluorescence intensity (MFI) remained largely unchanged, indicating that the majority of CD4^+^ T cells treated were still able to respond to early activation signals (Figure 2E). The MFIs of CD25 and IL-2 were significantly reduced (Figures 2F, G). (Shi et al., 2016) CD25 is an intermediate marker of T cell activation and is essential for their proliferation following TCR engagement (Boyman and Sprent, 2012), (Ross and Cantrell, 2018). In that case, the reduced expression of both IL-2 cytokine and its receptor, CD25, likely contribute to defective proliferation observed in the BI8626 treatment group. Similarly, sh-*Huwe1* did not inhibit CD69, but inhibited the expression of CD25 and IL-2 in these cells (Supplementary File S3A). Next, we detected the expression of phosphorylated ZAP (pZAP70), an important tyrosine kinase along the TCR signaling pathway, in CD4^+^ T cells upon activation of anti-CD3α (Figure 2H). Remarkably, we observed a decrease in the MFI of pZAP70 in both the BI8626 and sh-*Huwe1* groups, indicating a weakening of TCR signaling. These findings suggest that inhibition of *Huwe1* can impede the activation and proliferation of CD4^+^ T cells.

### 3.3 Huwe1-deficient CD4^+^ T cells have reduced cholesterol levels

The composition and arrangement of lipids in the plasma membrane dictate its “lipid order”, an essential factor for the correct localization of signaling proteins during the formation of immune synapses. Glycosphingolipids and cholesterol are the two important components of lipid order, and any alteration in their relative abundance and arrangement can significantly affect membrane protein interactions and signal transduction (Kidani et al., 2013), (Armstrong et al., 2010), (Bensinger et al., 2008).

We measured glycosphingolipid expression through fluorescently conjugated Cholera Toxin Subunit Beta (CTB), a well-established surrogate glycosphingolipid marker (Lencer and Tsai, 2003). After 24 h of αCD3/CD28 stimulation, CD4^+^ T cells treated with BI8626 showed no significant change in CTB fluorescence, indicating no alteration in glycosphingolipid concentration upon early activation (Figure 2I). In contrast, upon treatment with NB-DNJ (UDP-glucose ceramide glucosyltransferase inhibitor) to blockade glycosphingolipid synthesis, CTB fluorescence was significantly downregulated. This expression of glycosphingolipid remained unchanged until 48 h after activation (Figure 2I), suggesting that sphingolipid may not be directly associated with the inhibition of the early activation of BI8626 treated T cells. To further investigate this phenomenon, we used Fillipin III staining to quantify the cholesterol content of murine CD4^+^ T cells activated by anti-CD3 plus anti-CD28 antibodies. We found that both BI8626 and sh-*Huwe1* treatment decreased the amount of cholesterol (Figure 2J), which was further confirmed by total cholesterol detection (Figure 2K). Together, these findings indicate that inhibition of cholesterol content may be a potential mechanism underlying the suppression of CD4^+^ T-cell activation observed with BI8626/sh-*Huwe1* treatment.

### 3.4 Exogenous cholesterol supplementation enhances the activation and proliferation of Huwe1-deficient CD4^+^ T cells

**FIGURE 3:**
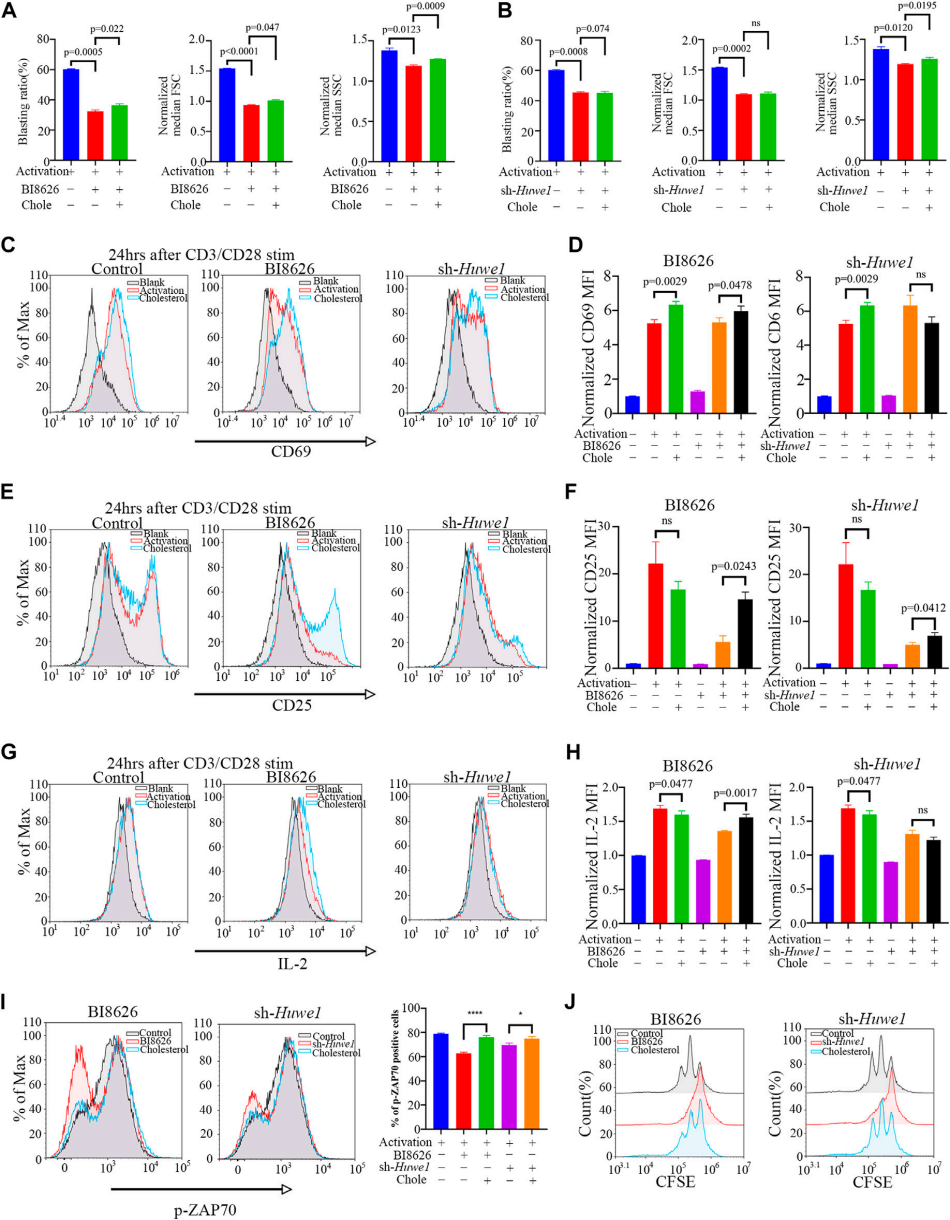
Exogenous cholesterol supplementation enhances the activation and proliferation of Huwe1-deficient CD4^+^ T cells **(A,B)** Effects of exogenous cholesterol on cell blasting of BI8626-treated **(A)** and sh-Huwe1-treated **(B)** CD4^+^ T cells was analyzed after 24h activation. Bar graphs represent mean ± SD (*n* = 3 mice/group from three independent experiments). **(C–I)** Rescuing effect of soluble cholesterol on BI8626-induced or siRNA-induced inhibition of CD4^+^ T cell activation (CD69 **(C,D)**, CD25 **(E,F)**, IL-2 **(G,H)**, p-ZAP70 **(I)**). Bar graph on right shows MFI±SD. **(J)** Rescuing effect of exogenous cholesterol on BI8626/sh-Huwe1-induced inhibition of CD4^+^ T cell proliferation. Exogenous supplementation of water-soluble cholesterol could improve the proliferation ratio of CD4^+^ T cells. Asterisk indicates *p* < 0.05, triple asterisks indicate *p* < 0.001 vs. control. Data are from one experiment representative of three **(A,B)**; (*n* = 3) or two **(D,F,H,I,J)**; (*n* = 2) independent experiments with biological duplicates in each.

Exogenously supplementing CD4^+^ T cells with soluble cholesterol (10μM, Sigma Aldrich), a methyl-β-cyclodextrin (MBCD)-conjugated form of the compound, can ameliorate defects in lipid homeostasis and improve cell survival (Hong and Amara, 2010), (Idowu and Hagenbuch, 2022), (Wilfahrt et al., 2021), (Marty-Roix et al., 2016), (Kidani et al., 2013). Interestingly, cholesterol was found to slightly rescue the blasting ratio, cell size, and cell granularity of CD4^+^ T cells treated by BI8626 (Figure 3A). Cholesterol had a certain effect on cell granularity in the sh-*Huwe1* group, but did not influence the blasting ratio and cell size (Figure 3B). There was no significant difference in the CD69 MFI between BI8626/sh-*Huwe1* treatment group and the control group, while the addition of cholesterol stimulated the upregulation of CD69 in the BI8626 treatment group (Figures 3C, D). Furthermore, the exogenous supplementation of soluble cholesterol was found to attenuate the suppressive impact of BI8626/sh-*Huwe1* on CD25 expression (Figures 3E, F). This approach also showed an obvious rescue effect on IL-2 expression of BI8626 treated CD4^+^ T cells (Figures 3G, H). Importantly, the exogenous administration of cholesterol improved p-ZAP70 levels after cell activation, indicating its potential to counteract the inhibitory effects of BI8626/sh-Huwe1 on TCR signaling intensity (Figure 3I). In addition, cholesterol also increased the proportion of proliferating CD4^+^ T cells (Figure 3J; Supplementary Files S3B, C). Thus, we posit that HUWE1/Huwe1 inhibition-mediated regulation of CD4^+^ T cell activation and proliferation is intricately linked to cholesterol, highlighting the importance of this metabolite.

### 3.5 *Huwe1*-deficient CD4^+^ T cells exhibited elevated levels of cholesterol efflux

**FIGURE 4:**
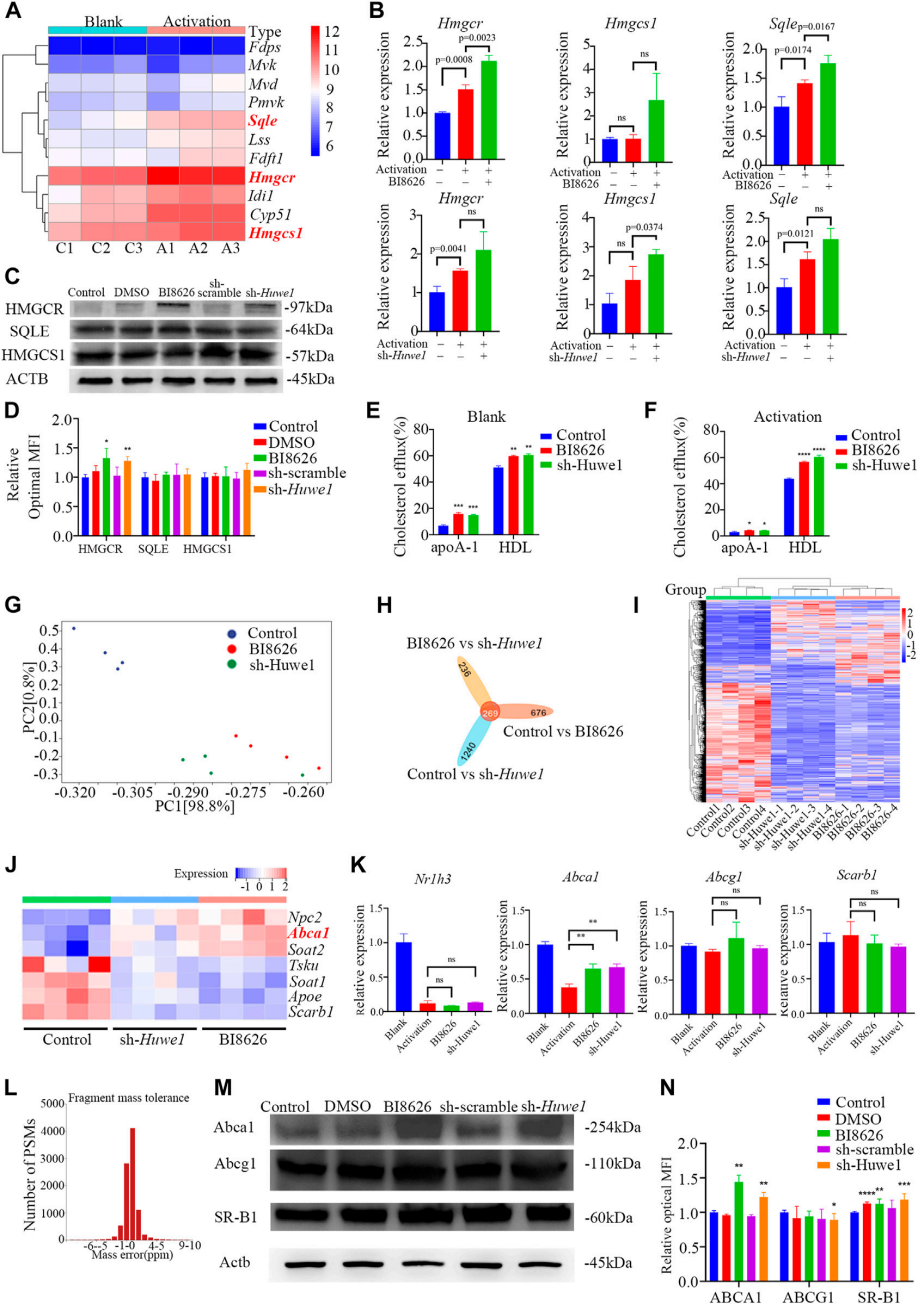
*Huwe1*-deficient CD4^+^ T cells exhibited elevated levels of cholesterol efflux **(A)** Heatmap of cholesterol biosynthesis-related enzyme genes expression in murine CD4^+^ T cells with or without activation. **(B)** Cholesterol biosynthesis-related enzyme gene expression was evaluated by real-time PCR. Expression of each gene was normalized to *β-actin* expression and normalized relative to expression in resting CD4^+^ T cells. Data shown are means ± SD. **(C,D)** Representative Western blot images **(C)** and quantitative analysis **(D)** of the expression of HMGCR, SQLE, and HMGCS1 in activated (anti-CD3ε and anti-CD28) murine CD4^+^ T cells in the indicated groups. Asterisks indicates *p* < 0.05, double asterisks indicate *p* < 0.01 vs. control. **(E,F)** Cellular cholesterol efflux to Apo AI (10 μg/mL) or HDL (50 μg/mL) from BODIPY-cholesterol loaded CD4^+^ T cells in the indicated groups. Asterisks indicates *p* < 0.05, double asterisks indicate *p* < 0.01, triple asterisks indicate *p* < 0.001, quadruple asterisks indicate *p* < 0.0001 vs. control. **(G)** Principal component analysis (PCA) plot of the mRNA-seq samples. **(H)** Venn diagram of the intersection DEGs of three different groups. **(I)** Heatmap of genes expression of all genes in murine CD4^+^ T cells in the indicated groups. **(J)** Heatmap of genes expression of cholesterol efflux-related proteins in murine CD4^+^ T cells with or without activation. **(K)** The gene expression of cholesterol efflux-related proteins was evaluated by real-time PCR. Expression of each gene was normalized to β-actin expression and normalized relative to expression in resting CD4^+^ T cells. Data shown are means ± SD. Double asterisks indicate *p* < 0.01 vs. control. **(L)** The deviation of peptide mass number conforms to normal distribution. **(M)** Western blot assay was conducted to test protein levels of ABCA, ABCG1, and SR-BI in activated CD4^+^ T cells in the indicated groups. **(N)** Quantitative analysis of protein expression of ABCA, ABCG1, and SR-BI in anti-CD3/CD28 simulated CD4^+^ T cells in the indicated groups. Asterisks indicates *p* < 0.05, double asterisks indicate *p* < 0.01, triple asterisks indicate *p* < 0.001 vs. control. Data are from one experiment representative of three independent experiments with similar results **(D,N)** or three independent experiments with biological duplicates in each **(B,E,F,K)**; (*n* = 3).

Based on our previously published sequencing data (Yin et al., 2022c), we found that the expression of key genes involved in *de novo* cholesterol synthesis was significantly increased after the activation of murine CD4^+^ T cells (Figure 4A). To confirm whether the inhibition of HUWE1/*Huwe1* affects the cholesterol synthesis pathway, we detected the expression of several relevant genes. Intriguingly, we found that BI8626 and sh-*Huwe1* treatments did not inhibit the mRNA levels of *Hmgcr*, *Hmgcs1*, or *Sqle* (Figure 4B). WB analysis revealed a significant increase in the protein levels of HMGCR in CD4^+^ T cells treated with BI8626/sh-*Huwe1* (Figures 4C, D). This may be caused by the combination of upregulation of HMGCR transcription and increased stability of HMGCR due to sterol deficiency (Leichner et al., 2011). The expression of other key rate-limiting enzymes involved in cholesterol biosynthesis remained relatively stable upon *Huwe1* inhibition (Figures 4C, D). Combined with the fact that the cellular cholesterol content of BI8626/sh-*Huwe1* group was upregulated in response to the CD3/CD28 stimulation (Figure 2K), we believe that *Huwe1* inhibition does not affect cholesterol biosynthesis. Therefore, we tested the cholesterol efflux ability of CD4^+^ T cells. Both BI8626 and sh-*Huwe1* treatments dramatically enhanced apo AI-mediated and HDL-mediated cholesterol efflux from CD4^+^ T cells, as evidenced by the results shown in Figures 4E, F. These findings suggest that the inhibition of Huwe1 may stimulate active cholesterol efflux, ultimately reducing intracellular cholesterol levels. It is known that activated T cells typically inhibit cholesterol efflux to maintain stable intracellular cholesterol levels, thereby facilitating further proliferation and effector differentiation (Michaels et al., 2021). Additionally, we also found that the mRNA expression levels of key cholesterol efflux transporters ABCA1, ABCG1, and NR1H3 in CD4^+^T cells of SS patients were significantly lower than those from healthy controls (Supplementary File S3D), suggesting that a weakening of cholesterol efflux function of CD4^+^ T cells is involved in the pathophysiology of SS.

We performed transcriptome sequencing on spleen CD4^+^ T cells from various groups of mice and found that the expression profiles of BI8626 and sh-*Huwe1* groups were similar (Figures 4G–I). Notably, our results reveal a significant upregulation in mRNA expression of *Abca1* upon treatment with BI8626/sh-*Huwe1* (Figure 4J). However, our analysis did not reveal any statistically significant differences in the expression of *Abcg1 and Scarb1* under the tested experimental conditions. We utilized RT-qPCR to verify the expression of several cholesterol efflux-related genes in murine CD4^+^ T cells. Specifically, we observed a significant upregulation in the mRNA expression of *Abca1* in both BI8626 and sh-*Huwe1* groups, while there was no difference in the expression of *Abcg1* and *Scarb1* (Figure 4K). Since the main function of HUWE1 is to regulate ubiquitinosomal degradation of proteins, we performed mass spectrometry (MS) to identify the proteins expressed in CD4^+^T cells. The fragment mass tolerance measured by MS conformed to a normal distribution (Figure 4L). Unfortunately, the abundances of ABCA1, ABCG1 and SR-B1 in murine CD4^+^ T cells were not high enough to be detected by the mass spectrometer (Supplementary File S4). Our WB results showed that both BI8626 and sh-*Huwe1* could significantly upregulate the expression of ABCA1 and SR-B1 proteins (Figures 4M, N), suggesting that E3 ubiquitin ligase HUWE1 might regulate the stability of ABCA1 and SR-B1.

### 3.6 ABCA1 could be ubiquitinated by HUWE1 and degraded by a proteasome

**FIGURE 5:**
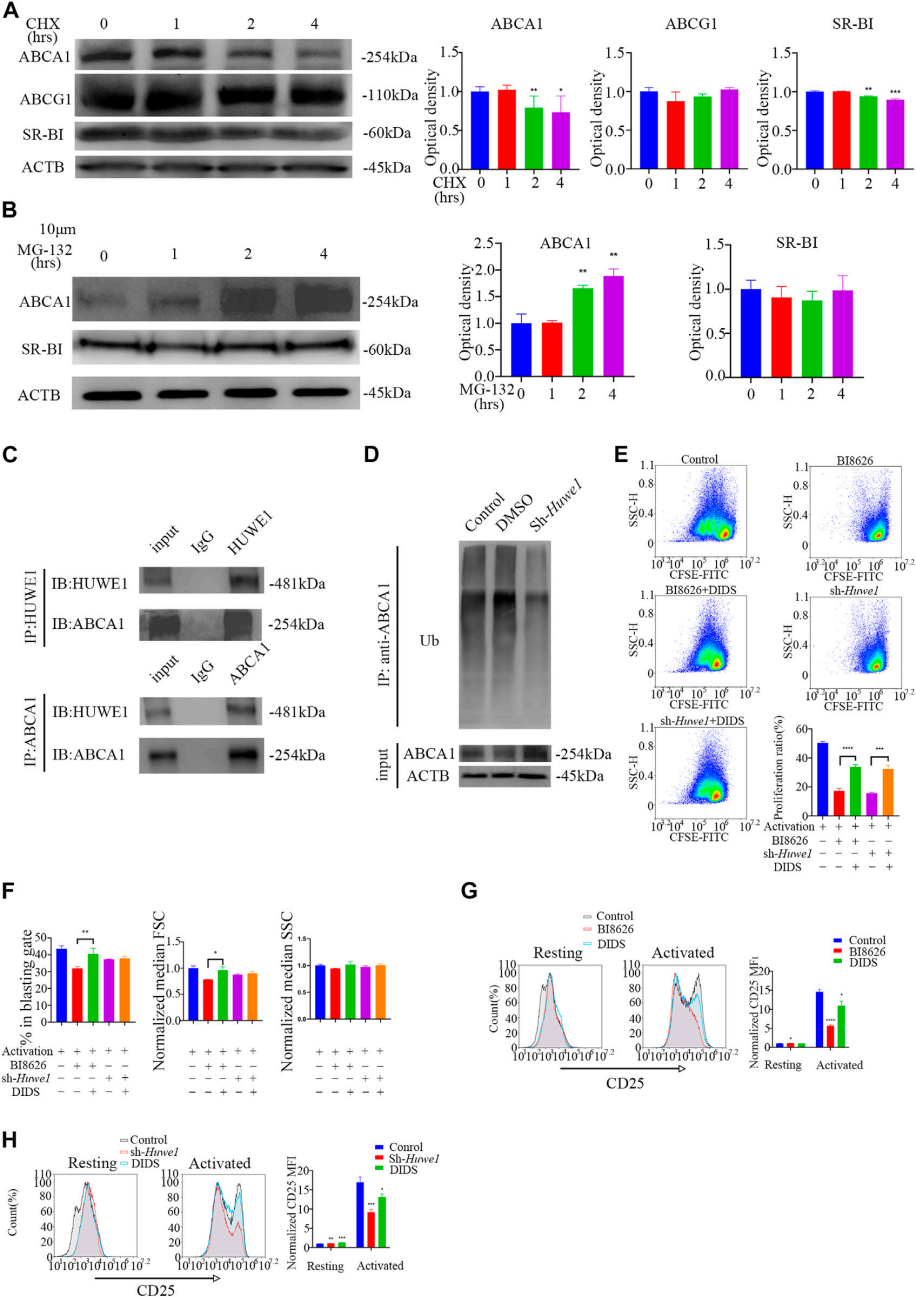
ABCA1 could be ubiquitinated by HUWE1 and degraded by proteasomes **(A)** The cells were treated with cycloheximide for the indicated times, and the expression of ABCA1, ABCG1, and SR-BI was analyzed by Western blotting. The expression of ABCA1 and SR-BI decrease over time. Double asterisks indicate *p* < 0.01, triple asterisks indicate *p* < 0.001 vs. control. **(B)** The expression level of ABCA1 in activated CD4^+^ T cells was restored by MG132 treatment. The cells were treated with 10 μM of MG132 for the indicated times, and the expression of ABCA1 and SR-BI was analyzed by Western blotting. The expression of ABCA1 increases over time. Double asterisks indicate *p* < 0.01 vs. control. **(C)** The interaction of HUWE1 with ABCA1 in CD4^+^ T cells was detected by co-immunoprecipitation assay. **(D)** Less ubiquitinated ABCA1 was observed in sh-Huwe1 treated cells than in control cells. The activated CD4^+^ T cells in the indicated groups were treated with MG132 and were then lysed and immunoprecipitated with anti-ABCA1. The enriched proteins were analyzed by Western blotting with anti-ubiquitin antibodies. **(E)** The proliferation of cells in the indicated groups was detected by CFSE staining. Bar graph on right shows the proliferation ratio of CD4^+^ T cells in different groups. Triple asterisks indicate *p* < 0.001, quadruple asterisks indicate *p* < 0.0001 vs. control. **(F)** Cell blasting of CD4^+^ T cells in the indicated groups was analyzed by size (forward scatter area, FSC) and granularity (side scatter area, SSC) after 24h activation. Asterisk indicates *p* < 0.05, double asterisks indicate *p* < 0.01 vs. control. **(G)** to **(H)** The CD25 expression in BI8626**(G)**- and sh-Huwe1**(H)**-treated CD4^+^ T cells was restored by DIDS. Bar graph on right shows MFI±SD (*n* = 3 mice/group from three independent experiments). Data are from one experiment representative of three independent experiments with similar results **(A–D)** or three independent experiments with biological duplicates in each **(E–H)**; (*n* = 3).

Activated CD4^+^ T cells treated with cycloheximide (CHX) for 8 h showed profoundly reduced expression of ABCA1 and SR-BI proteins, suggesting that both may be subject to proteasome degradation in early activation (Supplementary File S5A). However, the levels of ABCA1 and SR-BI proteins gradually recovered over time, potentially due to a weakening of inhibition by CHX. No significant change in ABCG1 expression was observed after 8 h of treatment. To further understand the degradation of these proteins during early activation, protein levels were measured within 4 h and revealed a gradual decrease in ABCA1 and SR-BI expression, while ABCG1 expression remained unchanged (Figure 5A). Treatment of cells with the proteasome inhibitor MG-132 revealed that ABCA1 protein levels increased over time (Figure 5B), indicating that proteasome-mediated degradation is responsible for the reduction in ABCA1 expression. In contrast, no significant change in SR-BI levels was observed, suggesting that its decreased protein levels are likely due to other degradation mechanisms. Co-immunoprecipitation assays in activated CD4^+^ T cells revealed an interaction between HUWE1 and ABCA1 (Figure 5C). Although we also tested for a potential interaction between HUWE1 and ABCG1/SR-BI, we found no evidence of this in our analysis (Supplementary File S5B). Subsequently, a ubiquitin pull-down assay in activated CD4^+^ T cells showed that knocking-down *Huwe1* decreased the ubiquitination of ABCA1 (Figure 5D). Collectively, these results suggest that HUWE1 interacts with ABCA1, diminishing its stability by mediating its ubiquitination-driven degradation, thus suppressing ABCA1 protein levels.

To confirm that HUWE1 depends on ABCA1 to regulate activation and proliferation, we treated CD4^+^ T cells with DIDS, a selective inhibitor of ABCA1. We observed that DIDS markedly increased the proliferation rate of CD4^+^ T cells treated with BI8626/sh-*Huwe1* (Figure 5E). The blasting ratio and cell size of CD4^+^ T cells treated by BI8626 were rescued by DIDS (Figure 5F). In addition, DIDS also restored the expression of CD25, which had been reduced by BI8626/sh-*Huwe1* (Figures 5G, H), suggesting that HUWE1 regulates T-cell activation through controlling the stability of ABCA1.

### 3.7 BI8626 exhibits a protective effect against the symptoms of spontaneous SS in NOD/ShiLtj mice

**FIGURE 6:**
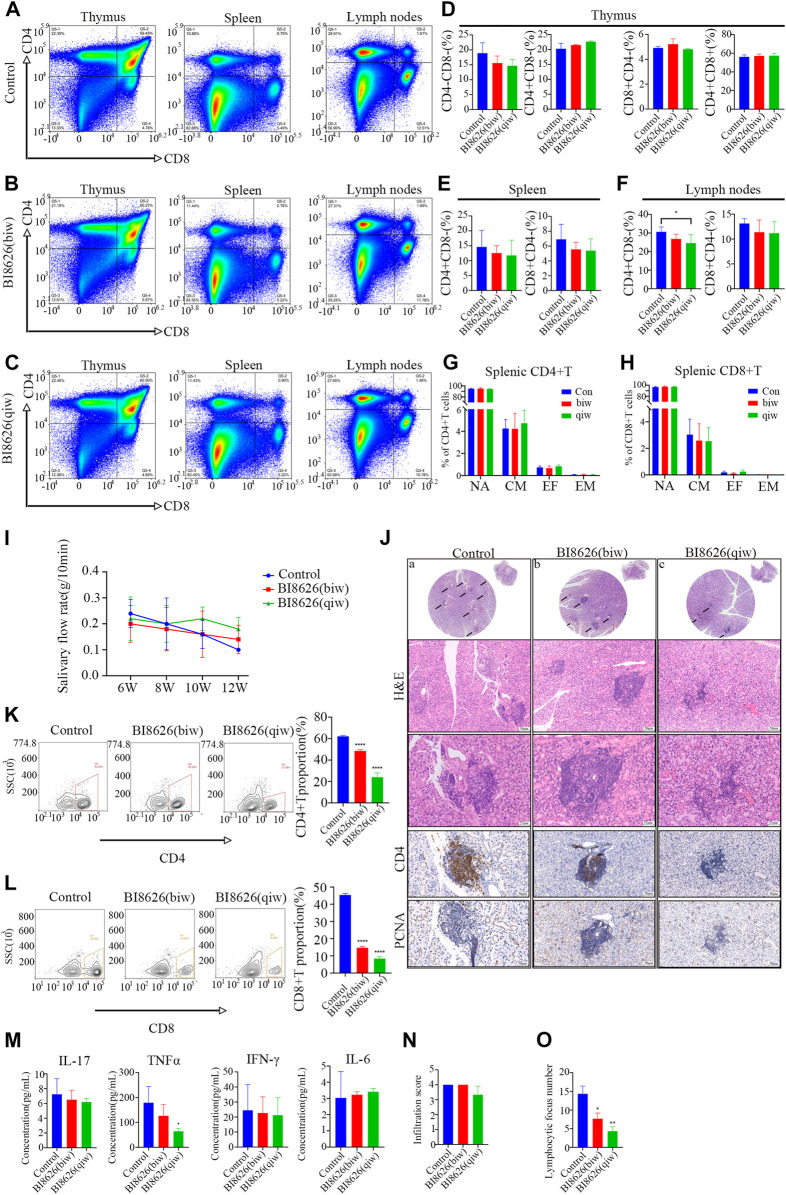
BI8626 exhibits a protective effect against the symptoms of spontaneous SS in NOD/ShiLtj mice **(A)** to **(C)** Representative dot plots showing CD4 and CD8 expression on thymocytes, splenocytes and lymphocytes to assess proportions of double-positive (CD4^+^CD8^+^) and single-positive (CD4^+^CD8^−^ or CD8^+^CD4^−^) (Gated on CD3^+^CD45^+^) in the indicated groups. **(D–F)** Quantitation of proportions of double-positive and single-positive cells in thymus **(D)**, spleen **(E)**, and lymph nodes **(F)**. Asterisk indicates *p* < 0.05 vs. control. **(G,H)** Mean frequencies of CD44^−^CD62L^+^ naïve, CD44^+^CD62L^+^ central memory, CD44^+^CD62L^−^ effector memory and CD44^−^CD62L^−^ effector T cells in splenic CD4^+^(G) or CD8^+^(H) T cell compartment. (*n* = 3 mice/group from two independent experiments). **(I)** Simulated salivary flow rate of NOD/ShiLtj mice treated with BI8626 at different dosages. The salivary flow rate was measured at the ages of 6, 8, 10, and 12 weeks. **(J)** Representative H&E staining and Immunohistochemical staining of CD4-positive cells and PCNA-positive cells in the salivary glands in the indicated groups. **(K,L)** Flow cytometric plots of lymphocytes [CD3^+^CD4+T cells **(K)** and CD3^+^CD8+T cells **(L)**] from salivary gland tissues of NOD mice with or without treatment. Proportion of CD4+T cells and CD8+T cells in salivary gland tissues of 12-week-old female NOD mice with or without treatment analyzed by flow cytometry. Quadruple asterisks indicate *p* < 0.0001 vs. control. **(M)** ELISA quantification of IL-17, TNF-α, IFN-γ, and IL-6 levels in circulating sera of the indicated NOD/Ltj mice. Asterisk indicates *p* < 0.05 vs. control. **(N)** The disease progression scores between the indicated groups. (*n* = 3 mice/group from two independent experiments). **(O)** Numbers of lymphocyte foci (lymphocyte numbers>50) between the indicated groups. (*n* = 3 mice/group from two independent experiments). Data are from one experiment representative of two independent experiments with biological duplicates in each **(D,E,F,G,H,I,K,L,M,N,O)**; (*n* = 5).

To assess the potential impact of targeting HUWE1 on SS, we conducted *in vivo* experiments using NOD/ShiLtj mice, a validated SS animal model. Nod/ShiLtj mice showed a large number of lymphocyte infiltrates in the submandibular gland, with CD4^+^ T cells forming the majority (Supplementary File S5C). Such locally infiltrated CD4^+^ T cells are usually overactivated (Fu et al., 2020). The disease phenotype in NOD/ShiLtj mice, including abnormal exocrine gland function and pathological changes, is analogous to the clinical manifestations of SS. To test the effect of BI8626 on thymocyte development, we evaluated the proportions of the major thymocyte populations (double-positive, CD4 single-positive, and CD8 single-positive cells) and found no significant changes (Figures 6A–D). No significant alteration in the proportions of single-positive CD4 and CD8 T cells in the spleen was observed following BI8626 treatment (Figure 6E). In contrast, we observed a slight decrease in the proportion of CD4^+^ T cells in the cervical lymph nodes (Figure 6F), indicating that local inflammation may have been ameliorated. Analysis of CD44 and CD62L expression revealed comparable frequencies of naïve (CD44^−^CD62L^+^), central memory (CD44^+^CD62L^+^), effector memory (CD44^+^CD62L^−^) and effector (CD44^−^CD62L^−^) T-cell subsets between control and BI8626-treated mice (Figures 6G, H). These findings suggest that BI8626 does not significantly alter the composition of the peripheral T-cell compartment. We examined SFR in NOD/ShiLtj mice with and without treatment on a weekly basis. Submandibular glands were isolated for HE staining, as previously described (Fu et al., 2020). In the control group, SFR decreased significantly over time; however, this reduction was partially counteracted in the BI8626-treated group (four times a week) (Figure 6I). This suggests that BI8626 treatments may ameliorate SS progression. Furthermore, histological analysis through HE staining showed a lower lymphocyte infiltration in the BI8626-treated (four times a week) group compared to the controls (Figure 6J). Immunohistochemical staining revealed a marked reduction in CD4^+^ T cells within the salivary gland lymphocyte infiltration foci of BI8626-treated NOD/ShiLtj mice, compared to the control group (Figure 6J). This was accompanied by a decrease in both CD4^+^ and CD8^+^ T cells (Figures 6K, L). Moreover, BI8626 treatment resulted in a significant decrease in the number of proliferating cells (PCNA positive) within the lymphocyte infiltration foci (Figure 6J), indicating a suppression of CD4^+^ T-cell proliferation. Collectively, these findings demonstrate that BI8626 suppresses CD4^+^ T-cell accumulation and proliferation in the submandibular glands of NOD/ShiLtj mice, providing a mechanistic explanation for the observed attenuation of symptoms. Additionally, we compared the serum concentrations of TNFα, IL-17, IFN-γ, and IL-6 between the control and BI8626-treated (four times a week) groups, and found that TNFα was decreased in the latter group (Figure 6M). While the resulting infiltration score was not significantly different (Figure 6N), the lymphocyte focus number in both BI8626-treated groups was significantly reduced compared to the controls (Figure 6O). Collectively, these results demonstrate that BI8626 treatments may be beneficial in ameliorating SS progression. These results suggest that HUWE1 inhibition can reduce disease progression by downregulating the activity and numbers of CD4^+^ T cells. Thus, our findings provide a rationale for the therapeutic potential of BI8626 in SS.

## 4 Discussion

Sjögren’s syndrome (SS) is an autoimmune disorder characterized by the infiltration of exocrine glands and an associated high lymphocytic infiltration. In labial gland biopsy samples of SS patients, a large number of lymphocyte infiltrates can be observed, releasing inflammatory factors that can impair the structure and function of salivary glands (Yao et al., 2021). Within these invaded lymphocytes, the majority are activated CD4^+^ T cells, which underscores the importance of studying the overactivation of CD4^+^ T cells in SS.

Previous studies have demonstrated that CD4^+^ T cells require substantial energy and metabolites to support their functioning after activation (Shan et al., 2020). At rest, they primarily rely on oxidative phosphorylation and fatty acid oxidation for energy. Upon activation, they exhibit a metabolic reprogramming characterized by increased reliance on glycolysis and glutamine metabolism. In our previous study, we showed that both CD4^+^ T-cell activation and Th17 differentiation depend on the glycolytic metabolic pathway, and that the application of the glycolytic inhibitor 2-DG could effectively reduce the infiltration of CD4^+^ T cells in the submandibular gland of NOD/ShiLtj mice (Fu et al., 2020). In our recent study, we showed that the pro-inflammatory function of CD4^+^ T cells was associated with the glutamine metabolic pathway and that inhibition of glutaminase 1 alleviated SS-like symptoms in NOD/ShiLtj mice (Fu et al., 2022). Notably, ingested glucose and glutamine not only provide energy to cells, but also serve as a source of carbon for cholesterol synthesis by T cells. Cholesterol in the plasma membrane is a key component of lipid rafts and is essential for the transmembrane transport and signal transduction of immune cells (Norata et al., 2015), (Luo et al., 2020), (O’Neill and Pearce, 2016). The transcriptional factor sterol regulatory element binding protein 2 (SREBP2) and two rate-limiting enzymes, 3-hydroxy-3-methylglutaryl-CoA reductase (HMGCR) and squalene monooxygenase (SQLE), regulate *de novo* cholesterol biosynthesis. Upon activation, SREBP2 promotes cholesterol synthesis in CD4^+^ T cells (DeBose-Boyd and Ye, 2018). In addition, interference with cholesterol synthesis in T cells has been shown to impede both the proliferation and activation of these cells in response to TCR signals (Kidani et al., 2013), (Kidani et al., 2013). Our previous study has shown that blocking *de novo* cholesterol synthesis by targeting cytochrome P450, family 51 (*Cyp51*) inhibits the activation and proliferation of both CD4^+^ and CD8^+^ T cells (Yin et al., 2022c). Meanwhile, pharmacological inhibition of cholesterol synthesis alleviated symptoms and reduced CD4^+^ T cell infiltration in salivary glands of SS-like mice. Similar to macrophages, T cells take up low-density lipoprotein (LDL) containing cholesterol esters via LDL receptors and obtain free cholesterol by hydrolyzing cholesterol esters through the lysosome system. Following stimulation with anti-CD3/CD28, CD4^+^ T cells synthesize cholesterol and reduce cholesterol efflux to preserve newly generated cholesterol in preparation for cell proliferation. Activation of the transcription factor LXR, a key regulator of cholesterol homeostasis, has been shown to increase the transcription of ABCG1, decreasing T-cell proliferation (Bensinger et al., 2008). Conversely, *Abcg1* knockdown in CD4^+^ T cells has been associated with significantly upregulated proliferation upon activation (Michaels et al., 2021), (Armstrong et al., 2010). Esterification, catalyzed by Acyl coenzyme A-cholesterol acyltransferase (ACAT1), is a process by which excess cholesterol is transformed into cholesterol esters and stored in cells as lipid droplets (Luo et al., 2020). Schmidt et al. and Yang et al. demonstrated that inhibition of this esterification process leads to a reduction in lipid droplets and an increased localization of cholesterol to the plasma membrane (Yang et al., 2016), (Schmidt et al., 2021). Cholesterol homeostasis has been found to not only affect the TCR nanocluster formation, signal transduction and differentiation, but also regulate the anti-infective ability of CD4^+^ T cells. Jiang et al. found that retinoic acid can promote ABCA1-mediated cholesterol efflux, thereby reducing the number of HIV-infected CD4^+^ T cells (Jiang et al., 2012). These findings underscore the importance of cholesterol homeostasis as a critical metabolic checkpoint in CD4^+^ T cells.

In this study, we showed that HUWE1 inhibition or Huwe1 knockdown in CD4^+^ T cells decreased the levels of both blasting and proliferation in response to activation. We further demonstrated that this effect is partially dependent on reduced intracellular cholesterol levels, as exogenous cholesterol supplementation improved cellular blast and proliferation. HUWE1 plays an important role in biological processes including cell proliferation, apoptosis, DNA damage responses, and inflammatory responses. HUWE1 has been found to cooperate with TRAF6 to form K48-K63 branched ubiquitin chains, thereby inhibiting the clearance of K63 by CYLD and enhancing the activity of NF-κB and the downstream expression of inflammatory genes (Ohtake et al., 2016). HUWE1 regulates the ubiquitination of AIM2, NLRP3 and NLRC4, supporting inflammasome activation (Guo et al., 2020). In addition, HUWE1 ubiquitinates Ets-1 for proteasomal degradation in CD4^+^ T cells, leading to a reduction in the number and functionality of Tregs (Li et al., 2021). To further elucidate the regulatory role of HUWE1 in cholesterol homeostasis in CD4^+^ T cells, we tested the expression of several rate-limiting enzymes in the cholesterol synthesis pathway and found no significant changes. Previous studies have indicated that HUWE1 is involved in post-translational modification of the cholesterol transporters ABCG1 and ABCA1 (Aleidi et al., 2015). To corroborate this, we tested the cholesterol efflux ability of CD4^+^ T cells treated with BI8626/sh-*Huwe1*, and observed that the inhibition of HUWE1 significantly enhanced the level of cholesterol efflux induced by Apo-AI and HDL. In activated CD4^+^ T cells, HUWE1 inhibition led to a marked upregulation of *Abca1* mRNA expression, but had no significant effect on *Abcg1* and *Scarb1*. At the same time, ABCA1 and SR-BI proteins were upregulated, while ABCG1 protein abundance was slightly decreased. It is likely that the upregulation of Abca1 transcription was caused by the decreased expression of ABCG1 protein, in a compensatory manner, to regulate cholesterol efflux in T cells (Zhao et al., 2022). During cholesterol efflux, HDL core ApoAI accepts cholesterol excreted by ABCA1, while HDL particles (including pre-β-HDL, α-HDL2, and α-HDL3) accept cholesterol from SR-BI and ABCG1 (Rozhkova et al., 2021), (Linton et al., 2017). In that case, the upregulation of ABCA1 and SR-BI proteins explains the upregulation of ApoAI and HDL-mediated cholesterol efflux levels. Furthermore, we found that the stability of ABCA1 protein is influenced by the ubiquitin-proteasome system, and that HUWE1 is the ligase that regulates ABCA1 ubiquitination. Although degradation of the SR-BI protein also occurs, this degradation is not mediated by the ubiquitin-proteasome. In our Co-IP assays, we showed that HUWE1 and SR-BI did not bind to each other, suggesting that there may be other degradation pathways of SR-BI. Taken together, these findings suggest that activated CD4^+^ T cells need to upregulate HUWE1 to post-translationally regulate ABCA1 protein levels to inhibit cholesterol efflux.

Currently, immunosuppressants such as glucocorticoids and chloroquine are commonly used clinically to treat SS (Saraux et al., 2016). However, long-term high doses of these drugs are associated with adverse effects, such as osteoporosis, maculopapule, and eye movement disorders. Therefore, efficacious drugs with favorable adverse effect profiles are urgently needed to improve the quality of life of SS patients. To explore the therapeutic effect of HUWE1 inhibition on SS, we intraperitoneally injected NOD/ShiLtj mice with the HUWE1 selective inhibitor BI8626. We found that BI8626 did not alter the T lymphocyte populations in the thymus, spleen, and lymph nodes, indicating that the drug had no adverse effect on the development of immune organs. Four times a week administration was associated with a better effect on the salivary flow rate, compared to the two times a week regimen. This may be related to the clearance rate of BI8626 *in vivo*; more frequent administration likely maintains a critical plasma concentration (Peter et al., 2014). BI8626 treatment decreased the level of TNFα in the peripheral blood of mice, and also decreased the number and infiltration score of lymphocyte infiltration in the submandibular glands of mice. The number of CD4^+^ and CD8^+^ T cells in the submandibular glands decreased, suggesting that inhibiting HUWE1 shows a promising therapeutic effect on SS-like symptoms.

In conclusion, we showed that HUWE1 can inhibit cholesterol efflux by regulating ABCA1 ubiquitination in activated CD4^+^ T cells to support cell proliferation. Meanwhile, inhibition of HUWE1 function alleviated SS-like symptoms in NOD/ShiLtj mice, which may provide a new direction for the treatment of SS.

## Figures and Tables

**Table:** 

**ABCA1**	ATP-binding cassette transporter A 1
**ABCG1**	ATP-binding cassette transporter G 1
**BP**	Biological process
**CDK2**	Cyclin-dependent kinase 2
**CDK4**	Cyclin-dependent kinase 4
**CFSE**	5-(and −6) -Carboxyfluorescein Daicetate Succinimidyl Ester
**DEGs**	Differentially expressed genes
**DMSO**	Dimethyl sulfoxide
**FDPS**	Farnesyl pyrophosphate synthase
**FBS**	Fetal bovine serum
**GEO**	Gene Expression Omnibus database
**GO**	Gene Ontology
**GSEA**	Gene Set Enrichment Analysis
**H&E**	Hematoxylin & eosin
**HMGCR**	Hydroxymethylglutaryl-CoA reductase
**HMG-CoA**	3-hydroxy-3-methyl glutaryl coenzyme A reductase
**IFN-γ**	Interferon-γ
**IL-17**	Interleukin-17
**IL-2**	Interleukin-2
**HMGCR**	Hydroxymethylglutaryl-CoA reductase
**IDI1**	Isopentenyl pyrophosphate isomerase 1
**MBCD**	Methyl-β-cyclodextrin
**MCL-1**	Myeloid cell leukemia-1
**mRNA**	Messenger RNAs
**NCBI**	National Center for Biotechnology Information
**PBS**	Phosphate buffered saline
**PCR**	Polymerase chain reaction
**PBMC**	Peripheral blood mononuclear cell
**SEM**	Standard deviations of the mean
**SFR**	Salivary flow rate
**siRNA**	Small-hairpin RNA
**SLE**	Systematic lupus erythematosus
**SQLE**	Squalene epoxidase
**SREBP2**	Sterol-regulatory element binding protein2
**SS**	Sjögren’s syndrome
**Th1**	T helper 1
**Th17**	T helper 17
**TNF-α**	Tumor necrosis factor-α

## Data Availability

The transcriptome sequencing data in this study are publicly available on the Sequence Read Archive (SRA). These data can be found here: SRP444253.

## Supplementary material

SUPPLEMENTARY FILE S2 The association between HUWE1 expression and activated CD4^+^T cells **(A)** Immunochemical staining of CD4-positive cells and HUWE1-positive cells in the labial glands of SS patients. **(B)** Lymphocyte infiltration was detected by H&E staining of the labial salivary glands of patients with pSS. **(C)** Transfection efficiency was evaluated by transfection indicator **(D)** Optical intensity of HUWE1 in murine CD4^+^ T cells following treatment with different shRNAs. Triple asterisks indicate p < 0.001, quadruple asterisks indicate p < 0.0001 versus control. **(E)** Representive flow cytometry images of Zombie-NIR and CFSE-based fluorescence intensity in the indicated groups. Compared with the control group, the number of apoptotic cells in BI8626 treatment group and sh-Huwe1 treatment group did not change significantly, but the level of cell proliferation changed significantly **(F)** Cell cycle distribution was detected with Propidium Iodide (PI) staining. Distribution of G0/G1, S, and G2/M phases in anti-CD3/anti-CD28-stimulated CD4^+^ T cells in the indicated groups. **(G)** Optical intensity of CDK2 in murine CD4+T cells in the indicated groups. Data are from one experiment representative of three independent experiments with similar results **(D,G)** or two independent experiments with biological duplicates in each **(E,F)**; (*n* = 3).

SUPPLEMENTARY FILE S3
*Huwe1* maintains the activation of CD4^+^ T cells **(A)** Enriched CD4^+^ T cells were stimulated with anti-CD3ε and anti-CD28 in the presence or absence of sh-Huwe1 and analyzed for CD69, CD25 and interleukin-2 expression after 24 h. Bar graph on right shows MFI±SD (*n* = 3 mice/group from three independent experiments). **(B,C)** Representive flow cytometry images of CFSE-based fluorescence intensity in the indicated groups. Cholesterol can ameliorate the inhibitory effect of BI8626 or sh-Huwe1 on cell proliferation. **(D)** Expression of ABCA1, ABCG1, SCARB1 and NR1H3 in circulating CD4^+^ T cells obtained from 5 healthy individuals and 11 SS patients. The expression of each gene was normalized to β-ACTIN expression. Three independent experiments were performed for qRT-PCR assays.

SUPPLEMENTARY FILE S5 Correlation between HUWE1 and cholesterol efflux transporter **(A)** The cells were treated with cycloheximide for the indicated times, and the expression of ABCA1, ABCG1, and SR-BI was analyzed by Western blotting. Asterisk indicates p < 0.05, quadruple asterisks indicate p < 0.0001 vs. control. **(B)** The interaction of HUWE1 with ABCG1 or SR-BI in CD4^+^ T cells was detected by co-immunoprecipitation assay **(C)** Lymphocyte infiltration was detected by H&E staining and immunohistochemical staining of the submandibular salivary glands of 6 (left) and 12 week-old (right) NOD/ShiLtj mice. Data from a single experiment, which is representative of three independent experiments with similar results, are presented **(A,B)**.
