# Supplementary figures and images for: HECT, UBA and WWE domain containing 1 represses cholesterol efflux during CD4+ T cell activation in Sjögren’s syndrome
Source: Front Pharmacol. 2023 Jun 26;14:1191692. doi: 10.3389/fphar.2023.1191692 (PMC10330700; doi:10.3389/fphar.2023.1191692)

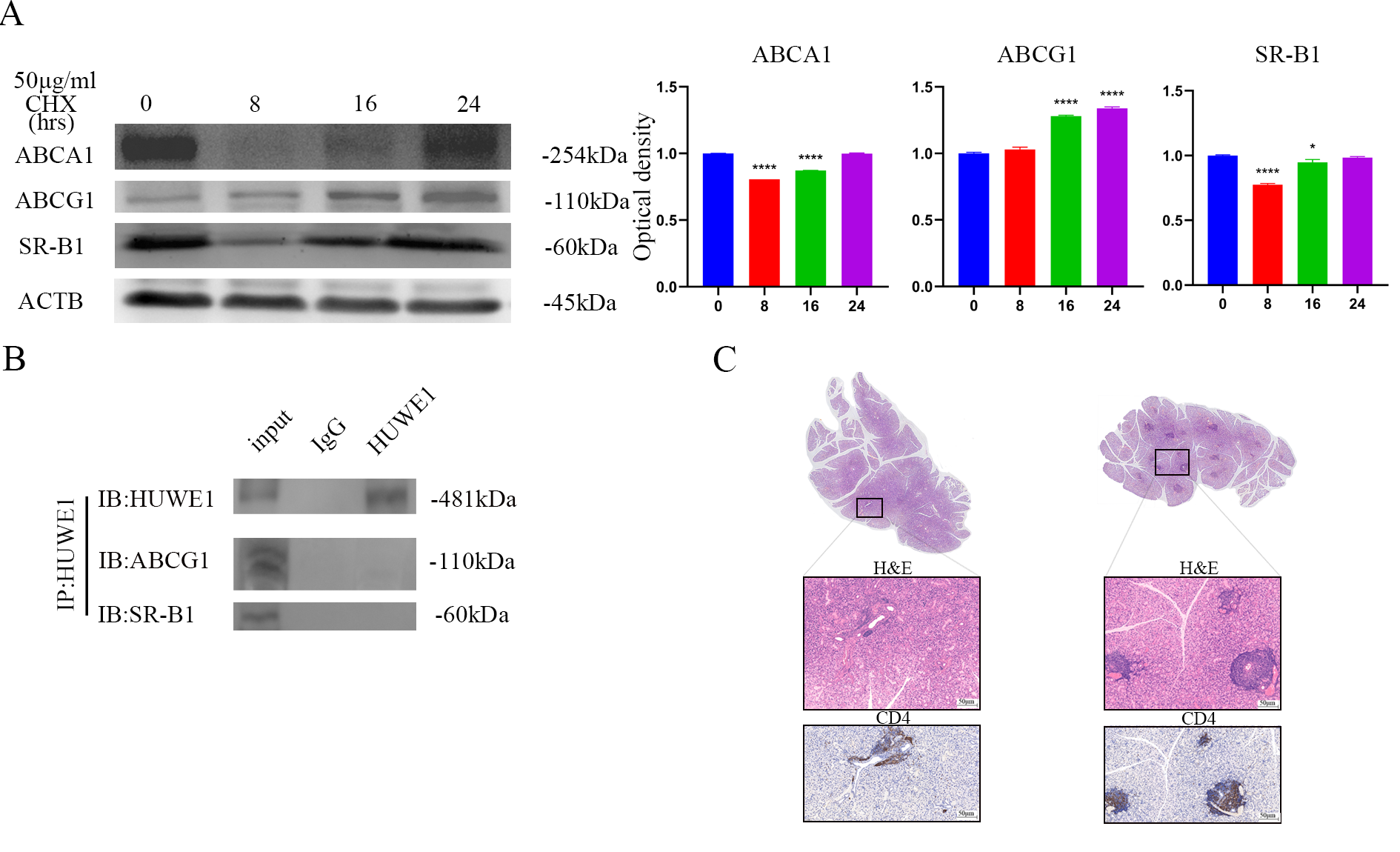

Supplement: Supplementary file 2 [file Image3.TIF]

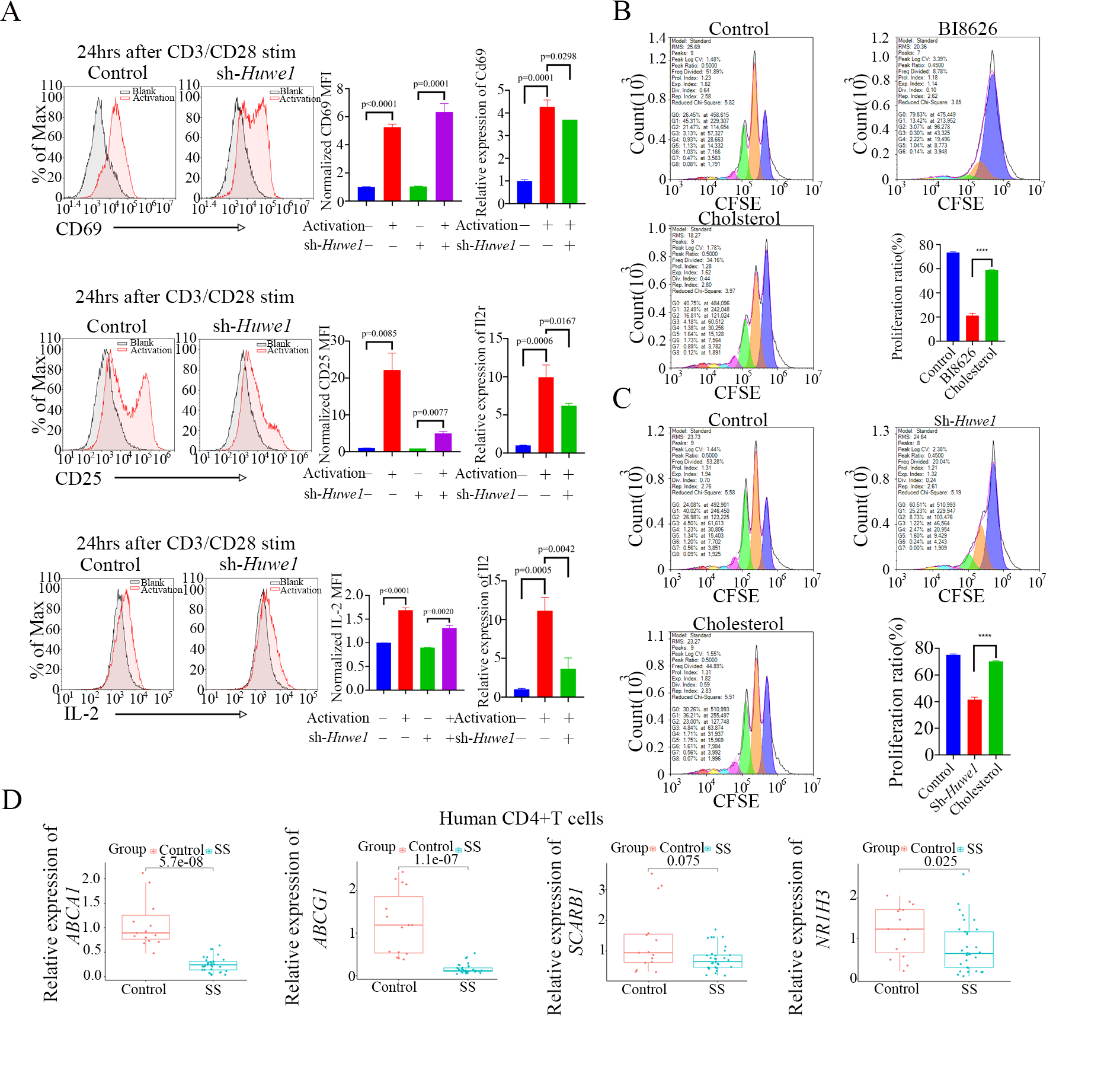

Supplement: Supplementary file 3 [file Image2.TIF]

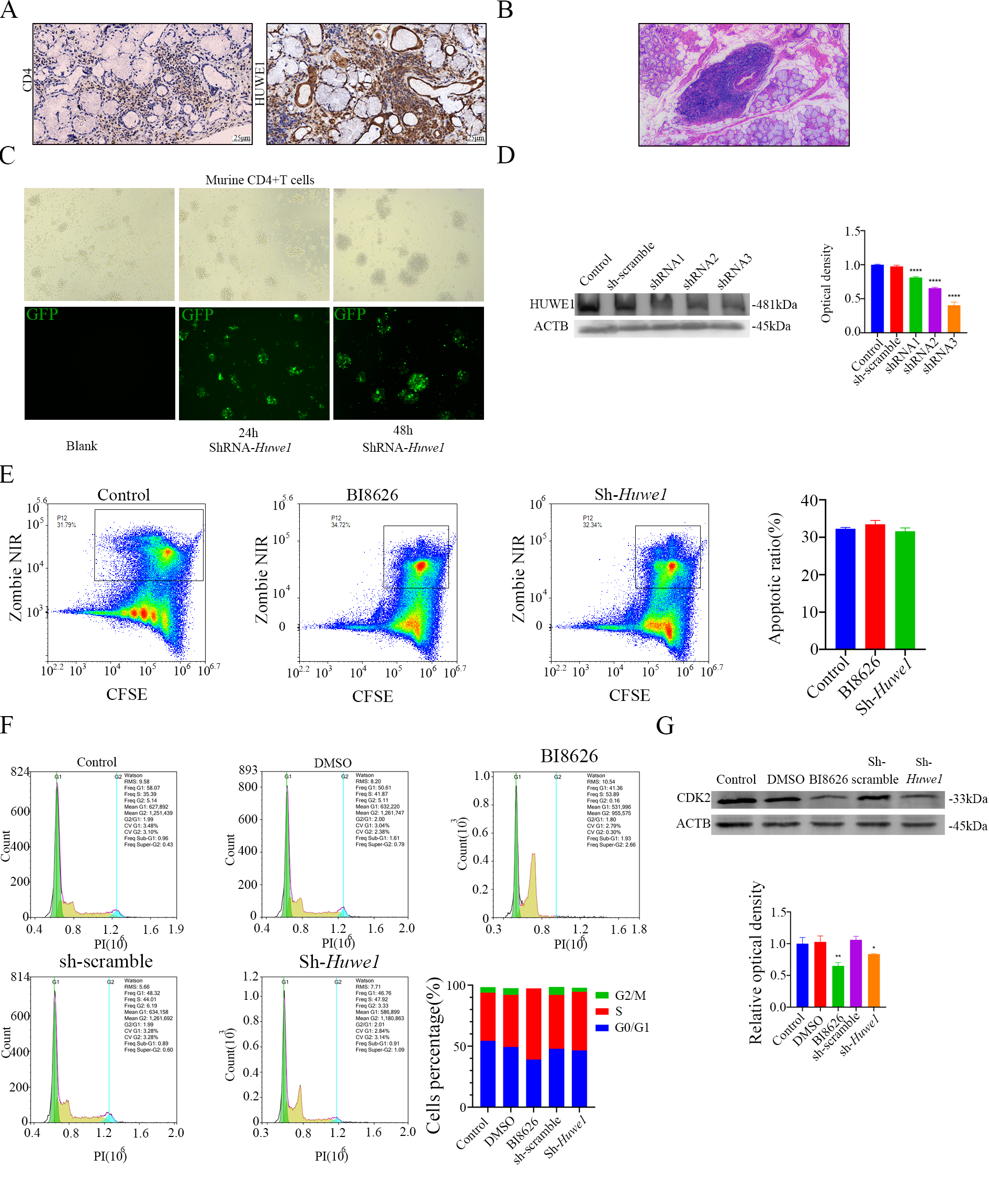

Supplement: Supplementary file 4 [file Image1.TIF]
